# Reading metacomprehension of Spanish deaf and hard-of-hearing students

**DOI:** 10.1093/jdsade/enae030

**Published:** 2024-08-12

**Authors:** Isabel R Rodríguez-Ortiz, Francisco J Moreno-Pérez, David Saldaña

**Affiliations:** Department of Developmental Psychology and Education, University of Seville, Seville, Spain; Department of Developmental Psychology and Education, University of Seville, Seville, Spain; Department of Developmental Psychology and Education, University of Seville, Seville, Spain

**Keywords:** deaf, reading, metacomprehension, inference-making

## Abstract

Difficulties in monitoring reading comprehension result in poor comprehension. One key aspect of monitoring is metacomprehension, which refers to one’s awareness of one’s own reading comprehension. Previous studies have observed difficulties in metacomprehension among the deaf or hard-of-hearing (DHH) population. This study aims to determine whether the metacomprehension of DHH students corresponds to their reading score and whether they are truly capable of adjusting their metacomprehension to the difficulty of the text. We evaluated 25 Spanish-speaking DHH students with reading scores approximately equivalent to Grades 5 or 6 of Primary School. Participants were asked to read a text and answer questions. The texts corresponded to three levels of difficulty (explicit, inferable, and noninferable). The results revealed that the metacomprehension of DHH students corresponded to their reading score. The DHH population may have better reading metacomprehension than is typically assumed, although the manifestation of this skill may depend on the type of task demanded of them (comprehension judgment or knowledge judgment).

Deaf and hard-of-hearing (DHH) children frequently encounter challenges in understanding written text (see Harris, 2015, for a review). As part of the effort to explain these difficulties, the simple view of reading (SVR; Gough & Tunmer, 1986; Hoover & Gough, 1990) has inspired many studies on reading comprehension among this population. According to this model, reading comprehension is the product of two sets of skills: decoding and linguistic comprehension. Decoding refers to the ability to recognize written words and linguistic comprehension refers to the ability to understand spoken language at a discursive level. The use of this model tries to understand the reading difficulties experienced by the DHH population, and has given rise to many studies focusing on exploring both the role of decoding and the variables associated with it (Deng & Tong, 2021; Dodd, 1987; Domínguez et al., 2014; 2019; Hanson, 1989; Hanson et al., 1991; Hayes & Arnold, 1992; Kyle & Harris, 2011; Paul, 1998, 2003; Trezek et al., 2010; Wang et al., 2008, 2018), and the role of linguistic comprehension (Moreno-Pérez et al., 2015; Rodríguez-Ortiz et al., 2017; Deng & Tong, 2021; Domínguez et al., 2016; Kyle & Harris, 2011; Miller, 2010, 2013; Miller et al., 2012, 2013; Quigley et al., 1977; Santamaría et al., 2018; Worsfold et al., 2018). However, recent studies have shown that other processes may also be involved in reading comprehension. Reading comprehension is conceptualized as the result of cognitive and linguistic factors that interact with both the reading goals and the nature of the texts being read (Nation, 2019), with the ultimate aim of enabling the reader to construct a mental model (or situation model in the terms used by Kintsch, 1988) that allows them to understand the text at a deeper level than merely its explicit content.

In this sense, Kim (2017) proposed the direct and indirect effect model of reading (DIER model), which integrates those skills identified in the SVR (word reading and listening comprehension), along with other linguistic and cognitive competencies (working memory, vocabulary, grammatical knowledge, inference, and comprehension monitoring) in a hierarchical model. In that model, comprehension monitoring, or, in other words, the ability to supervise one’s own reading comprehension, detect mistakes, and propose strategies to correct them, plays a key role. Specifically, in the DIER model, comprehension monitoring is believed to influence reading comprehension through listening comprehension. There is a large body of evidence attesting to the fact that deficits in comprehension monitoring are a trait that clearly distinguishes readers with poor reading comprehension (Oakhill et al., 2005). In the DIER model, comprehension monitoring is directly affected by basic language skills (vocabulary and grammar). Since DHH children often experience difficulties in both basic language skills (Bishop, 1983; Kelly, 1996; Kyle & Harris, 2010; Lillo-Martin et al., 1992; Musselman, 2000; Paul, 1996, see also Lederberg et al., 2013, for a review) and reading comprehension (Kyle & Cain, 2015; see also Harris, 2015, for a review), exploring comprehension monitoring in this population is of particular interest.

Monitoring skills form part of metacognition, defined as knowledge and self-regulation of one’s own cognitive processes (Flavell, 1976). Several studies have shown that metacognitive skills are what distinguish proficient comprehenders from poor comprehenders. Specifically, it has been observed that poor comprehenders have less accurate metacomprehension (awareness of their own comprehension) (Bransford et al., 1982), are less sensitive to the demands of the task (Wong et al., 1982), and have fewer strategies for resolving comprehension difficulties (Myers & Paris, 1978).

## Metacognition and reading comprehension in deaf individuals

Few studies have focused on metacognition in deaf adolescents (Banner & Wang, 2011), and fewer have explored its role in reading comprehension (Schirmer, 2003; Trezek et al., 2010). In studies focusing exclusively on the DHH population, it has been observed that DHH adolescents have difficulty in accurately assessing their metacognitive knowledge (Davey, 1987) and tend to overestimate their reading skills (McCarr, 1973).

Gibbs (1989) studied awareness of the problems affecting reading comprehension in a sample of 19 prelingually and profoundly deaf high-school students aged between 16 and 19 years, with very varied reading skills (4th to 12th grade equivalent level), enrolled at the California School for the Deaf and with Wechsler IQ scores above average (*M* = 115.9). Participants were asked to detect internal inconsistencies, knowledge violations, and nonsense words in the provided texts. The DHH students detected fewer than half of the comprehension problems they had. This skill correlated with their reading level (*r* (19) = .65; *p* < .01).

Subsequently, Strassman (1992) interviewed 29 prelingually and severe to profoundly deaf adolescents (average age 17 years, range 14;7–19;5 years), with average intelligence, and enrolled in a high school of a state residential school for the deaf, focusing on their metacognitive knowledge about school-related reading. Strassman (1992) observed that participants’ metacognition was incomplete. This was manifested in the fact that most claimed to seek assistance from other people if they had trouble understanding what they were reading, and only six participants said they used strategies such as re-reading the paragraph. Moreover, 42.86% said they thought the best way to answer a question about what they had read was to look for the words contained in the question in the text itself.

Kelly et al. (2001) conducted two studies, the first of which they focused on the comprehension of a scientific text by deaf college students, seeking to determine whether or not they were aware of their level of comprehension and were able to detect incongruent content information within the passage provided. Their sample in this study was composed of 20 students, most of them were between 18 and 25 years old, and all of them had hearing parents. Participants were divided into two groups: 10 deaf college students representing the higher-level reading group (reading at ninth grade level) and 10 deaf college students representing the lower-level reading group (reading at eighth-grade level). In comprehension tasks, both groups performed equally poorly, only about 50% of the participants in each group were able to identify the main ideas of the passages and fewer than 25% of them adequately responded to questions about the texts. Furthermore, 90% of the participants, that is, 100% in the lower-level reading group and 80% in the higher-level reading group, were unable to detect incongruent sentences. These results suggest a possible association between reading comprehension and monitoring skills among DHH students.

For their part, Banner and Wang (2011) compared the reading skills of five deaf adults (aged between 27 and 36 years) with those of six deaf students (aged between 16 and 20 years), all with profound prelingual hearing loss, and identified the strategies they used during reading by means of think-aloud questions during the reading process. The results revealed that only highly skilled readers were able to deploy metacognitive strategies, such as reading monitoring, and were consequently able to detect their own comprehension mistakes and use a variety of strategies to correct them. Poorer readers, on the other hand, often skipped unknown words or sentences without thinking about them and always used the same type of strategy, without paying attention to whether it was suitable for the task at hand. However, the most important differences between more and less skilled readers were related to their assessment of their own comprehension.

Salehomoum (2022) designed an intervention to analyze the effect of explicit instruction on reading comprehension strategies in a sample of four deaf students aged between 16 and 17 years. The results indicated a lack of consistent identification of unknown words and a lack of awareness of comprehension breakdowns. The author noted that a lack of awareness of comprehension difficulties is common among the DHH population.

One study that compared DHH students with their hearing counterparts in terms of these skills was conducted by Davey (1987), who studied the effect of type of question on reading comprehension and metacomprehension in a sample of 61 proficient hearing readers (age; 11 years), 62 poor hearing readers (age; 15 years), and 50 prelingually, profoundly deaf readers (age; 15 years); all with an equivalent reading level (between fifth- and sixth-grade levels). All deaf participants attended a residential school for the deaf and had become deaf prior to the age of 2 years. In this case, the metacomprehension of DHH students was not compared with that of a group of peers matched on chronological age. In relation to metacomprehension, in general, all three groups of readers tended to demonstrate more accurate perceived comprehension when they were allowed to reread the text than when they were not allowed to do so; however, DHH readers differed from the two groups of hearing readers in terms of the effect of the look back on their perception of their comprehension. Whereas hearing readers’ perception of their own comprehension was greater when they were allowed to reread the text, DHH readers were not aware of their better comprehension under this condition, despite the fact that their comprehension did in fact improve when they were permitted to reread the text.

In a series of four experiments, Borgna et al. (2011) compared the metacomprehension of DHH and hearing college students, examining their performance during both the reading of a text and attendance at a classroom lecture. In general, hearing college students made more accurate metacognitive judgments than DHH students, with overestimations more frequent than underestimations. DHH students were twice as likely to overestimate as to underestimate their performance, while hearing students underestimated and overestimated their performance with the same frequency. Content familiarity improved the accuracy of metacognitive judgments in both groups of students.

Similarly, Morrison et al. (2013) also studied metacomprehension in a sample of 109 DHH university students and 108 hearing university students during reading comprehension, using the Reading Awareness Inventory, which is an inventory that evaluates the metacognitive awareness of students about reading strategies. Parallel versions of this inventory were created to assess metacognitive awareness during the comprehension of a signed discourse (deaf students) and an oral discourse (hearing students). Metacognitive awareness of students with DHH was significantly correlated with their reading level (*r* (76) = .27), whereas this was not the case among their hearing peers.

Silvestri and Wang (2018) conducted a study analyzing psycholinguistic and sociocognitive facilitating factors for effective reading in signing adults who are profoundly deaf and do not use hearing technology. Their sample comprised four groups (15 deaf high-achieving, 15 deaf low-achieving, 14 hearing high-achieving, and 14 hearing low-achieving participants) classified in accordance with self-reported education levels and professional experience. The authors used a think-aloud discussion of reading strategies. In relation to the monitoring of text processing, the greatest differences were found in participants’ ability to recognize the source of comprehension problems, with the deaf high-achieving group scoring 11, the deaf low-achieving and hearing low-achieving groups scoring 9, and the hearing high-achieving group 13; whereas the frequency with which participants identified what they did or did not understand was similar among the deaf groups and the hearing high-achieving group (15–14), and somewhat lower for the hearing low-achieving group (12). However, these results should be taken with caution, since they are based on participants’ perceptions of their own ability to monitor reading comprehension and must be validated with other types of tests. They also refer only to signing deaf adults. Using the same sample and procedure, Wang et al. (2018) found a significantly positive relationship between reading comprehension and metacognitive skills, for both deaf and hearing participants.

The studies outlined above suggest that the DHH population tends to experience metacomprehension difficulties. Therefore, it is important to continue studying this skill in the DHH population, given its close relationship with reading comprehension. The present study aims to explore this question. Additionally, more research is required to determine whether the difficulties faced by the DHH population in terms of metacomprehension are associated with the lack of adequate exposure to a language, which frequently affects DHH people, and/or whether they are simply the result of a lower level of reading comprehension. For this reason, an additional sample of hearing children was added that was matched in reading score to the DHH participants, but the metacomprehension of each group was examined separately. The study also aims to analyze whether low metacomprehension manifests independently from the difficulty level of the text itself, with three levels being established: Texts with explicit information, texts with easily inferable information, and texts with information that is impossible to infer. Since no previous studies have been conducted on how adolescents adjust their metacomprehension to the different levels of difficulty of the texts, the present study includes a sample of hearing adolescents of chronological age matched with DHH participants, whose data are also analyzed separately. Specifically, the study aims to answer the following questions:

(1) Is the metacomprehension of DHH students related to their reading comprehension? We expect to find that their metacomprehension corresponds to their reading score.
(2) Are DHH students able to adjust their metacomprehension skills according to the difficulty level of the text? We expect to find more positive comprehension and knowledge judgments in relation to explicit texts and less positive ones in relation to noninferable texts, with inferable texts being located in an intermediate position.

## Methods

### Participants

The sample comprised three groups: A young severe-to-profound (more than 70 dB) prelingually deaf group (DHH group) (*n* = 25; 16 boys, 9 girls), a group of 25 hearing children and adolescents (14 boys, 11 girls) matched individually on a one-to-one basis with the first group in terms of chronological age (CA group) and a group of 25 hearing children and adolescents (13 boys, 12 girls) matched individually on a one-to-one basis with the DHH group in terms of reading score (RS group). Table 1 shows the characteristics of the sample. The three groups were matched on both gender (χ^2^(2) = .76, *p* > .05) and nonverbal IQ (WISC-IV or WAIS-IV) (*F*(2, 72) = 3.061, *p* > .05). The DHH and CA groups were equivalent in terms of age (*p* = .152) and education level (χ^2^(3) = 3.41, *p* = .182), with most of the participants having reached secondary education. The DHH and RS groups were equivalent in terms of reading score (*p* > .999), measured using the Reading Ability Test by Carrillo and Marín (2009) (see Instruments section), but participants in the DHH group were older than in the RS group (*p* = .009), who mostly were in primary schools. All participants had IQ scores within the normal range (*M_DHH_* = 93.36; *M_CA_* = 100.08; *M_RS_* = 100.40) and did not have any additional disabilities.

**Table 1:** Background data of participants.

	Deaf group (DHH group) (*n* = 25)	Chronological-age control group (CA group) (*n* = 25)	Reading-score control group (RS group) (*n* = 25)
Age [*M* (SD)]	13.63 (2.39)	14.97 (2.18)	11.58 (2.53)
Sex (boys/girls)	16/9	14/11	13/12
Education level^a^	8/14/3	3/16/6	16/8/1
Reading score [*M* (SD)]	40.24 (15.23)	50.68 (10.35)	39.64 (13.15)
Nonverbal IQ [*M* (SD)]	93.36 (11.18)	100.08 (11.06)	100.40 (11.73)
Oral receptive vocabulary (years) [*M* (SD)]	8.92 (2.81)	14.92 (2.17)	11.17 (3.14)

^a^Primary school/secondary school/higher education.

Table 2 shows the characteristics of the DHH group. All DHH participants attended mainstream schools in which oral Spanish was the primary means of communication. The speech perception abilities of the DHH group were low, according to the reports of their teachers and parents, despite using hearing aids and cochlear implants.

**Table 2:** Background characteristics of the DHH group (*n* = 25).

Background characteristics		*N*	% of *N*
Etiology of deafness			
	Genetic	3	12
	Illness	6	24
	Unknown	16	64
Level of hearing loss			
	Severe (>70 dB)	11	44
	Profound (>90 dB)	14	56
Hearing device			
	Hearing aid	16	64
	Cochlear implant (early cochlear implant, <4 years)	9 (1)	36 (4)
Communication mode			
	Speech	20	80
	Bilingual (speech and sign)	5	20
Onset of deafness			
	Prelingual (<3 years)	21	84
	Postlingual (≥3 years)	4	16

All participants were recruited through schools and associations of deaf in different cities in Spain. The authors were involved in the recruitment process by sending the consent form and information letter that the schools and associations provided to families interested in participating in the study.

This study was conducted in accordance with all ethical requirements and with the principles outlined in the Declaration of Helsinki of 1964. The Andalusian Regional Biomedical Research Ethics Board approved the recruitment and data collection procedures and signed and informed consent of all participants (or their legal guardians) was obtained before the start of the tests.

### Instruments

#### Reading efficacy

Reading efficacy was assessed using the Reading Ability Test (READ; Carrillo & Marín, 2009), which provides a global reading measure combining both comprehension and speed. Participants are given 64 sentences with the last word missing. Four response options are given underneath each sentence: The correct word and three distractors that are orthographically or phonographically similar to the correct response. Sentences are placed in increasing order of difficulty in terms of length, syntactical complexity, and word frequency. Participants must complete as many sentences as they can in 5 minutes. The raw score is the number of current answers (sentences correctly completed in 5 minutes) minus one third of the number of incorrect answers given. This measure has a high test–retest reliability, *r*(376) = .88, *p* < .001, and high internal reliability (Cronbach’s α = .96) (Cuadro et al., 2009).

#### Nonverbal IQ

Wechsler Intelligence Scales for Children or for Adults (WISC-IV o WAIS-IV; Wechsler, 2005, 2012) were administered to evaluate nonverbal IQ. The Cronbach’s alpha coefficients for the WISC-IV subtests in Spanish range from .70 to .90, and for the WAIS-IV from .87 to .97, indicating good internal consistency.

#### Oral receptive vocabulary

The Spanish Peabody Picture Vocabulary Test–TVIP (Dunn et al., 2006) was used to assess oral receptive vocabulary in Spanish. This instrument is considered a valid and reliable measure of vocabulary and general linguistic competence (Williams, 1999). The Cronbach’s alpha for this instrument is .90.

#### Metacomprehension task

The metacomprehension task was a researcher-developed task. Participants were given 18 texts (plus three additional texts at the beginning of the task for rehearsal purposes) pertaining to three different conditions: Six inferable texts (each text included a pseudoword; the meaning of the pseudoword could be determined by a clue given in a different sentence within the same text), six noninferable texts (with a pseudoword that could not be deduced from context) and six explicit texts (with no pseudowords). All participants were exposed to all three conditions, although they only read one condition for each text. For example, Subject 1 only read Text 1 in the inferable condition, Text 2 in the noninferable condition, and Text 3 in the explicit condition, and Subject 2 only read Text 1 in the noninferable condition, Text 2 in the explicit condition, and Text 3 in the inferable condition. Table 3 provides an example of texts from all three conditions.

**Table 3:** Example of the experimental reading metacomprehension assessment task.

Noninferable text	Inferable text	Explicit text
A boy was running through a meadow. He was tired, but decided to make an effort. Suddenly, the nagaro burst. They all looked at each other and decided to go home.	A boy was running across a football field. He was tired, but decided to make an effort. Suddenly, the nagaro burst. They all looked at each other and decided to go home.	A boy was running across a football field. He was tired, but decided to make an effort. Suddenly, the ball burst. They all looked at each other and decided to go home.
Comprehension judgment: How well did you understand the text? 1. Poorly... 4 Well		
Knowledge judgment: How well would you be able to answer a question about the text? 1. Poorly... 4 Well		
Inferential true or false question: All the children were scared when they saw the blood. True or False		

*Note*. The pseudoword in the noninferable and inferable conditions of this example is “nagaro” and the word that replaces it in the explicit condition is “ball.” The corresponding clue in the inferable condition is “football field,” which is replaced in the noninferable condition by the word “meadow.”

For each text, participants were asked to rate their level of comprehension (comprehension judgment) and their ability to answer a question about it (knowledge judgment). At the end of each text, participants were also asked an inferential true or false question about the content. One point was awarded for each correct answer (up to a maximum of 18), although the correct response rate for each of the individual conditions was used for the subsequent analyses. Half of the statements were true and half were false, and all were distributed evenly in terms of the sentence of the text to which they referred (25% of questions referred to the first sentence, 25% to the second, 25% to the third and 25% to the last sentence).

The task was programmed using E-Prime v.2.0. The first screen presented the complete text spread out over four lines. Participants pressed the space bar to move to the next screen, which contained the comprehension judgment question (how well did you understand the text?). Participants responded by pressing 1, 2, 3, or 4 to indicate whether they had understood poorly (1) or well (4), with two intermediate values close to poorly (2) and well (3). The next screen contained a knowledge judgment question (how well would you be able to answer a question about this text?). The participants responded using the same 4-point response scale (poorly to well). After pressing the corresponding key to answer the knowledge judgment question, participants moved to a screen containing an inferential true or false question about the text, which they were asked to answer by pressing V for true or F for false.

The global accuracy rate obtained in this task for the entire sample was correlated with the reading score of the READ test (*r(*75) = .361; *p* = .001) and other measures of reading speed (*r*(75) = .410; *p* < .001) and reading comprehension (*r*(75) = .361; *p* = .001) evaluated with the Reading and Writing test (TALE 2000; Toro et al., 2000). This is a standardized test for assessment of reading and writing skills widely used in Spanish. The Cronbach’s alpha for the full experimental task was .70.

### Procedure

Data collection was carried out in two individual sessions, each lasting approximately one hour, in a quiet and well-lit room in the schools of the participants. The evaluators were psychologists with experience as research technicians, who had been specifically trained by the authors to administer the tests and tasks, following the standardized procedures. The order in which the tasks were administered was counterbalanced. In all sessions, two evaluators were present, and 100% agreement was achieved between them through consensus during the administration of the tasks. Manual scoring protocols were used that included checks for scoring accuracy. The first author compiled and analyzed the results. This analysis was checked by the other authors.

## Results

The analyses were carried out using the SPSS 26 statistical package, using nonparametric tests because the data did not fulfill the application criteria for parametric tests as they were not normally distributed. In all cases, the raw scores of the instruments were used.

The data that support the findings of this study are openly available in Rodríguez-Ortiz et al. (2024) at https://doi.org/10.12795/11441/159577.

In relation to the first aim of the study (analyzing the metacomprehension of a sample of DHH students and determining whether it corresponded to their reading score), we conducted Mann–Whitney tests. Table 4 presents the descriptive statistics of the different groups.

**Table 4:** Reading comprehension monitoring in deaf and hearing students.

		Deaf group (DHH group) (*n* = 25)	Chronological-age control group (CA group) (*n* = 25)	Reading-score control group (RS group) (*n* = 25)
		*M* (SD)	*M* (SD)	*M* (SD)
Noninferable text	Success rate	.50 (.2)	.58 (.18)	.61 (.18)
	Comprehension judgment	2.94 (.56)	2.98 (.7)	3.22 (.48)
	Knowledge judgment	3.00 (.65)	2.98 (.62)	3.28 (.63)
Inferable text	Success rate	.61 (.25)	.79 (.14)	.76 (.17)
	Comprehension judgment	3.08 (.61)	3.2 (.50)	3.36 (.45)
	Knowledge judgment	3.06 (.58)	3.18 (.48)	3.38 (.58)
Explicit text	Success rate	.72 (.26)	.88 (.15)	.78 (.2)
	Comprehension judgment	3.32 (.59)	3.74 (.44)	3.56 (.55)
	Knowledge judgment	3.36 (.51)	3.7 (.38)	3.62 (.44)

As the DHH group was matched in terms of reading score with one of the hearing groups (RS), both groups scored similarly for all types of text. In other words, the success rate for both groups was similar for explicit, inferable, and noninferable texts. No differences were observed between the DHH group and the RS control group in terms of knowledge and comprehension judgments for the three types of text. Therefore, it can be concluded that the metacomprehension level of the DHH group corresponded to their reading score.

To respond to the second aim of the study, namely, to analyze whether DHH students were able to adjust their metacomprehension to the difficulty of the text, we compared their success rates and comprehension and knowledge judgments for the different types of text. We expected to find higher values in relation to explicit texts and lower values in relation to noninferable texts, with inferable texts being located in an intermediate position. To analyze the data, we performed a Friedman’s ANOVA, followed by the Wilcoxon signed rank test. In this case, a Bonferroni correction was applied, with all effects reported at a significance level of .017 (.05/3 comparisons between conditions).

In the DHH group, differences were observed between the three types of text in terms of success rate, *χ*^2^ (2) = 11.39, *p* < .05, although only the value for explicit texts was significantly higher than the value for noninferable texts, *z* = −1.65, *p* = .005, *r* = .19. The same result was observed in both the CA group [*χ*^2^ (2) = 22.56, *p* < .001; *z* = −3.971, *p* < .001, *r* = .56] and the RS group [*χ*^2^ (2) = 8.95, *p* < .05; *z* = −2.54, *p* = .011, *r* = .36], also in these two groups, participants performed better in the inferable than in the noninferable texts (*z* = −3.42, *p* = .001, *r* = .48, for the CA group and *z* = −2.95, *p* = .003, *r* = .42, for the RS group).

Regarding the judgment of the participants on their level of comprehension, differences were observed in the DHH group between the three types of text [*χ*^2^ (2) = 9.09, *p* < .05], although post hoc comparisons revealed that they were only significant between explicit and noninferable texts, *z* = −2.53, *p* = .011, *r* = .36. The same result was observed in both the CA group [*χ*^2^ (2) = 2.29, *p* < .001; *z* = −3.43, *p* < .001, *r* = .49] and the RS group [*χ*^2^ (2) = 14.31, *p* < .001; *z* = −2.50, *p* = .013, *r* = .35], also in the CA group, comprehension judgments were higher for explicit texts than for inferable texts, *z* = −3.50, *p* < .001, *r* = .49.

Knowledge judgments were higher for explicit than for noninferable texts in the DHH group [*χ*^2^ (2) = 12.12, *p* = .002; *z* = −2.95, *p* = .003, *r* = .42], the CA group [*χ*^2^ (2) = 27.36, *p* < .001; *z* = −3.81, *p* < .001, *r* = .54], and the RS group [*χ*^2^ (2) = 6.52, *p* = .038; *z* = −2.70, *p* = .007, *r* = .29]. In the DHH group also, the knowledge judgments were higher for explicit than for inferable texts, *z* = −2.66, *p* = .008, *r* = .38. The same result was also observed in the CA group, *z* = −3.64, *p* < .001, *r* = .48), although not in the RS group.

An additional measure of the reader’s adjustment to the difficulty of the text was provided by recoding the comprehension and knowledge judgment variables to obtain a measure of congruence for each. This enabled us to compare the congruence between groups. To this end, a new variable was created with one category corresponding to a congruent judgment and another to an incongruent judgment. Judgment was considered to be congruent when the overall success rate was at least 75% and responses 3 or 4 were chosen on the Likert-type scale that assessed the corresponding global judgment (comprehension or knowledge), or when the overall success rate was lower than 75% and responses 1 or 2 were chosen on that Likert-type scale. Since inferential responses were dichotomous, a 75% success rate was chosen to eliminate the effect of chance. Any other circumstance that did not meet these congruence criteria was classified as an incongruent judgment.

The distribution of congruent and incongruent comprehension and knowledge judgments in the three participant groups is shown in Table 5. The DHH group had a higher number of participants with incongruent comprehension judgments than the CA group, χ2(1) = 5.56, *p* < .05, although the difference with the RS group was not significant. In terms of knowledge judgment, although there were more participants with incongruent judgments in the DHH group than in the other groups, the differences were not statistically significant.

**Table 5:** Congruence of comprehension and knowledge judgments among the groups.

		Deaf group (DHH group) (*n* = 25)	Chronological-age control group (RA-group) (*n* = 25)	Reading-score control group (RS group) (*n* = 25)
Comprehension judgment	Congruent	5	13	8
	Incongruent	20	12	17
Knowledge judgment	Congruent	6	12	9
	Incongruent	19	13	16

In conclusion, DHH students’ performance in reading comprehension and monitoring skills was fairly similar to that of their reading score-matched hearing peers.

## Discussion

The present study was prompted by observations made in previous studies regarding the difficulties experienced by DHH students in terms of reading comprehension monitoring, with reports indicating a lower level of metacomprehension (Borgna et al., 2011; Morrison et al., 2013) and lower sensitivity toward the demands of the task (Davey, 1987), both variables that are associated with reading comprehension problems in the general population (Bransford et al., 1982; Wong et al., 1982), as well as in the DHH population (Morrison et al., 2013).

The study compared the performance of the DHH group with that of two hearing control groups, one matched on chronological age and the other of younger peers matched on reading score. Participants were given three types of text with different comprehension difficulty levels depending on whether they contained explicit, inferable, or noninferable information. In the last case, the objective was to check whether the participants were capable of adjusting their metacomprehension to the characteristics of the text being read.

Participants in the DHH group were matched in terms of reading score (measured using the READ test) with their hearing peers in the RS group. Consequently, their performance was similar in all three types of text used in the experimental task (explicit, inferable, and noninferable).

In the two hearing groups (CA and RS), the success rate was higher for inferable than for noninferable texts, a finding that may be considered an indication of their inference-making skills. In the DHH group, however, the success rate for inferable texts was similar to that of noninferable ones, suggesting that this group faced difficulties to make the necessary inferences to decipher the meaning of the pseudowords in the inferable texts. Consequently, their performance in inferable texts was no better than in noninferable ones. Previous studies comparing the inference-making skills of DHH students with those of their hearing peers (Doran & Anderson, 2003; Walker et al., 1998) have observed that DHH population tend to experience difficulties in this field, particularly when they have a lower reading score (Walker et al., 1998).

Regarding the first of the study’s objectives (to analyze metacomprehension of DHH students and detect whether it corresponds to their reading score), no differences were observed between DHH students and the RS group for any of the three types of text (explicit, inferable, and noninferable) in comprehension judgments (how well they had understood the text) or knowledge judgments (how well they could answer a question about the text). No differences were observed between these two groups in the number of participants with incongruent judgments. Therefore, the results indicate that the assessment of the DHH students of their own reading comprehension was equivalent to their level of reading.

Furthermore, unlike that observed in previous studies with adolescents or young adults (Borgna et al., 2011; Davey, 1987; McCarr, 1973; Morrison et al., 2013), the DHH participants in our sample were found to differ less from their peers matched on chronological age in terms of both comprehension and knowledge judgments. Indeed, the only differences observed between the two groups (DHH and CA) were for explicit texts (*U* (*N*_DHH group_ = 25, *N*_CA group_ = 25) = 181,5, *z* = −2,758, *r* = −.39), for comprehension judgement and *U*(*N*_DHH group_ = 25, *N*_CA group_ = 25) = 193, *z* = −2.469, *r* = −.35 for knowledge judgment, with (in contrast to that reported in previous studies) DHH students underestimating their comprehension, emitting lower comprehension and knowledge judgments than their counterparts in the CA group, despite not differing from them in terms of their actual comprehension of explicit texts. This was the main reason why there were more participants with incongruent comprehension judgments in the DHH group than in the CA group.

However, there were not significantly more participants with incongruent knowledge judgments in the DHH group than in the CA group, perhaps because the task involving answering a question about a text was very familiar to both groups, since this is an exercise they often carry out at school. In their study, Borgna et al. (2011) observed that content familiarity improved the accuracy of metacognitive judgments in both groups of students in their sample. In our case, rather than being familiar with the content of the task, it was familiarity with the type of task that may have helped the participants in the three groups make more accurate knowledge judgements. However, the results presented in Table 5 require us to be cautious when affirming this, since the difference in the number of participants with incongruent comprehension and incongruent knowledge judgments was very small.

The results obtained in the present study for the metacomprehension of DHH students, which are more similar to hearing readers than reported in previous studies, may be due to the fact that the reading score of DHH participants in our study was not as comparatively low as that of those participating in previous studies (Davey, 1987; Gibbs, 1989; Kelly et al., 2001; McCarr, 1973; Salehomoum, 2022; Strassman, 1992) because the reading scores of the DHH participants (mostly in secondary schools) were equivalent to those of the RS participants (mostly in the last grades of primary schools). Banner and Wang (2011) argue that what distinguishes more competent from less competent deaf readers is their ability to assess their own comprehension. For their part, Morrison et al. (2013) found that the metacomprehension of DHH students was correlated with their reading level. Consequently, better reading competence is usually accompanied by more accurate metacomprehension, and this may be the reason for the results found for the DHH group in our study.

In relation to metacomprehension, the data reported for DHH students in our study are consistent with the Qualitative Similarity Hypothesis (QSH; Paul & Lee, 2010), which posits the existence of a reading comprehension development trajectory that is common to both hearing students and DHH students, which would explain why DHH students’ metacomprehension corresponds to their level of reading.

Regarding the second aim, namely, to analyze whether DHH students are able to adjust their metacomprehension to the difficulty level of the text, their performance in terms of comprehension judgments was similar again to that of their reading score-matched peers. In both groups, comprehension judgments were more positive in the case of explicit than in the case of noninferable texts. This indicates that, at these reading scores and regardless of the presence or absence of deafness, individuals are aware of the difference between more and less difficult texts and adjust their assessment of their own comprehension accordingly, although not to the same extent as the participants in the CA group, who were also able to perceive the fact that inferable texts were more difficult than explicit ones. This finding also supports the QSH (Paul & Lee, 2010), insofar as DHH students adjust their metacomprehension to the difficulty of the text at hand to the same extent as their reading score-matched hearing peers.

However, in relation to knowledge judgments, DHH students performed similarly to CA students, being capable of perceiving that answering questions about explicit texts would be less difficult than answering questions about noninferable and inferable texts. Participants in the RS group, on the other hand, were only able to distinguish between explicit and noninferable texts, despite really responding better to questions about inferable texts than to questions about noninferable ones. As stated earlier, knowledge judgments by DHH students (and those in the CA group) may have benefited from their greater experience in the educational context; since being older than the participants in the RS group, they would have had more experience with this type of task (answering questions about texts), which may in turn have helped them calibrate their self-perceptions more accurately. According to Nietfeld et al. (2005), these types of judgment about one’s own performance depend on both the content of the task (text read) and the task itself (being required to answer a question).

The results outlined above suggest that the DHH population may have better reading metacomprehension that has commonly been attributed to them in previous studies and that this skill may manifest (or not) in accordance with the type of demand to which they are exposed (comprehension judgment or knowledge judgment). In this sense, and again as stated earlier, it is not just familiarity with the content that may affect this ability (Borgna et al., 2011), but also familiarity with the requirements of the task itself.

This observation may, however, lead us to overlook the fact that, despite everything, DHH students made more incongruent comprehension judgments than their counterparts in the CA group, indicating that their metacomprehension level is lower than would normally be expected in light of their age and school level. This metacomprehension difficulty is consistent with that observed in relation to DHH high school students’ knowledge of vocabulary (Krinsky, 1990), sign language comprehension (Marschark et al., 2005), and reading comprehension (Banner & Wang, 2011; Borgna et al., 2011; Davey, 1987; Gibbs, 1989; Kelly et al., 2001; McCarr, 1973; Morrison et al., 2013; Salehomoum, 2022; Strassman, 1992). In all these cases, the predictions of the DHH students about their own performance did not correspond to the final scores obtained. This lower metacognitive capacity has been associated not only with less proficiency in the specific academic skill at play here, but also with a lack of early exposure to a linguistic and educational environment that stimulates development (Borgna et al., 2011). In our study, we controlled for academic level, and participants in the CA group were matched to those in the DHH group for this variable. However, the level of oral language was lower in the latter group (DHH students had an equivalent age of 8.92 years in terms of oral receptive vocabulary, their speech perception skills were low, according to the reports of their teachers and parents, and only five of them used sign language.) Consistently with that reported by Maki et al. (2005), our data may, therefore, support the role of language in metacomprehension.

Another possible explanation for the lower level of metacomprehension observed among DHH students can be found in studies comparing poor comprehenders with good comprehenders in terms of comprehension monitoring. Paris and Myers (1981) and Garner and Kraus (1981), for example, suggested that the two groups have different reading goals, with poor comprehenders tending to pay more attention to decoding words than to understanding the meaning of the text, resulting in a lower level of comprehension monitoring. The lower reading scores of the DHH students in our sample put them in a situation similar to that of poor comprehenders.

The differences observed between DHH students and their peers matched on chronological age in terms of metacomprehension and inference skills draw attention to the need for the former group to receive explicit instruction in those skills that hearing students acquire incidentally, since these same skills have been associated not only with better reading competence, but also with academic success. Therefore, DHH students need to be exposed to texts that are complex enough to allow them to apply these skills. Nevertheless, according to Strassman (1997) and Marschark et al. (2002), all too frequently DHH students are exposed only to simplified reading materials. With the use of text that are complex enough and explicit instructions, the DHH students could be encouraged to answer inferential questions (i.e., why did this happen?), to make (and revise) predictions based on the title, subheadings, etc., to connect what they are reading to their previous knowledge, to use the context cues to infer the meaning of unknown words. And to enhance the reading metacomprehension, the DHH students could be taught to make pauses during their reading to ask themselves whether they are understanding what they are reading, to identify mistakes in a text, to summarize what they are reading, to self-evaluate their comprehension after reading a text and their ability to answer questions about it, to answer what part of the text was more difficult to understand, why and what type of strategies did they use to understand it, to discuss with others their different interpretations of the same text, among other activities.

## Limitations and future directions

The present study has a number of limitations that should be taken into account. The most obvious is related to the sample size, although it is important to note that the control groups used were equivalent to the DHH group in terms of chronological age (CA group) and reading age (RS group). Second, the texts used in the experimental task were short and not particularly difficult, which may have resulted in less marked differences between DHH students and their peers of the same age. However, the texts used did serve to highlight the difficulties experienced by DHH students in relation to inference making, along with the fact that their metacomprehension performance was similar to that of their reading-score-matched peers.

Future studies should strive to continue to analyze the specific metacognitive strategies associated with competent reading, exploring in more depth the causes of any possible deficits observed in these strategies among deaf students. Only in this way, we can design intervention measures that are truly adapted to the needs of this population. Indeed, the observation that the reading metacomprehension of deaf adolescents is similar to that of their peers of the same reading scores suggests that it may be possible to provide DHH students with instructions in these processes to allow them to reach the same level as their peers of the same age. It also suggests that there may be a reciprocal relationship between reading scores and the development of monitoring skills that future longitudinal and intervention studies may wish to explore further.

## Conclusion

The present study has provided information on the metacomprehension abilities of DHH students. Contrary to previous research that highlighted significant metacomprehension difficulties among DHH students, our findings suggest a more nuanced reality. When matched for the level of reading, DHH students demonstrated metacomprehension abilities comparable with those of their hearing counterparts, indicating that this skill is aligned with their actual reading proficiency.

Deaf students were able to adjust their metacomprehension to the difficulty of the text being read, like their reading-score-matched peers, in relation to comprehension judgments, and as their chronological age-matched peers, when judging their ability to answer questions about the text already read. So, the type of demand to which they are exposed (comprehension judgment or knowledge judgment), together with their experience in the educational context, could contribute to the performance in reading metacomprehension.

However, despite these promising results, the DHH group did exhibit more incongruent comprehension judgments compared to their peers matched on chronological age, reflecting an underlying challenge to accurately monitor their comprehension. This discrepancy underscores the need for targeted instructional strategies to improve metacomprehension and inference-making skills among DHH students. Such skills are crucial for their academic success and can be cultivated through exposure to more complex reading materials and explicit teaching methods.
